# A Manual of Operative Surgery

**Published:** 1900-12

**Authors:** 


A MANUAL OF OPERATIVE SURGERY. By LEWIS A. STIMSON, B. A., M. D., Professor of Surgery in Cornell University Medical College. New (4th) and thoroughly revised edition. In one royal 12mo volume of 581 pages with 293 illustrations. Just ready. Philadelphia and New York: Lea Brothers & Co. 1900. [Price, cloth, $3.00 net.]

The fourth edition of this well known little work has been brought fully up to date. The author has enlarged the section containing conclusions drawn from personal experience and has omitted portions of the text and a number of illustrations, which in his judgment have lost their usefulness. The book covers the entire field of operative surgery, even including sections on the eye, ear and female generative organs, and yet it is of convenient size, owing to the concise and compact style of description. Part I. is devoted to the accessories of an operation, the preparation of patient, instruments and sutures, and we mark with pleasure that anesthesia by nitrous oxide gas and ether is given favorable mention. In the chapter on excisions of bones and joints the author gives his own operation for old unreduced Potts' fracture.

The chief value of the book lies in the practical points preceding the description of each operation, or series of operations, the surgical anatomy of the parts, the originator of the operation with bibliographical reference, the indications for choice of operation and hints as to technique, which experience only can afford. Altogether we can commend author and publisher on the result obtained.—P. LEB.

